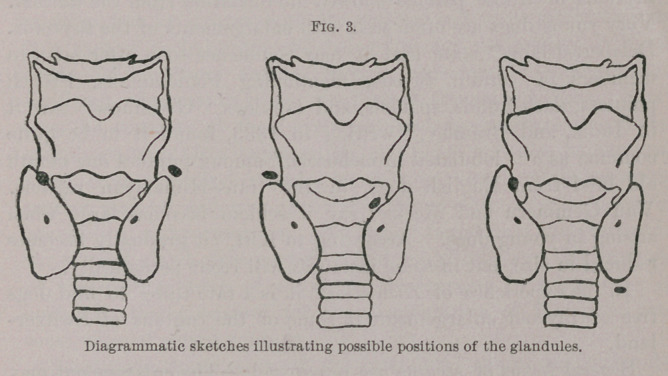# The Thyroid Gland and Thyroid Glandules of the Dog

**Published:** 1901-01

**Authors:** Cecil French

**Affiliations:** Washington, D.C.


### THE JOURNAL OF COMPARATIVE MEDICINE AND VETERINARY ARCHIVES.

Vol. XXII. *JANUARY, 1901.* No. 1.

72

### THE THYROID GLAND AND THYROID GLANDULES OF THE DOG.

By Cecil French, D.V.S.,
WASHINGTON, D.C.

### *The Thyroid Gland.*

Gross and Minute Structure. A well-developed organ consisting of two lobes. Each lobe presents an internal and external face, an anterior and posterior border, a rounded superior extremity, and a somewhat pointed inferior extremity forming the so-called cornua. They are connected in the larger breeds by a pronounced isthmus, which is irregularly present in medium-sized breeds, and invariably absent in the smaller breeds (Ellenberger and Baum[1]). They are situated in a position lateral to the trachea, immediately beneath the larynx, and are covered laterally by the sternocleidomastoideus muscles. In intimate relationship are the carotid and superior and inferior thyroid arteries, the internal jugular vein, and the vagus, sympathetic, recurrent laryngeal, and first cervical nerves.

Examined microscopically, each lobe is seen to possess a fibrous capsule, from which trabeculæ of connective tissue are projected inwardly to form a framework or stroma. The stroma subdivides the organ into lobules which support a number of cell clusters or vesicles. The stroma is traversed by numerous bloodvessels and lymphatics, and the walls of the vesicles are supplied with a rich capillary and lymphatic network. The lymphatics form a dense system, consisting of anastomosing vessels, spaces, and canals, traversing the gland in all directions. Baber[2] observed that the vesicles were very much branched in young animals, and he regarded this as a process of division of the vesicles by involution of their walls. They are lined with epithelium, which are columnar

180147

in the early stages, but become cubical later. Their lumina may or may not contain colloid matter. A homogeneous mass resembling morphologically that contained in the vesicles can be seen in the lymphatics. Both Baber[2] and Horsley[3] held that the function of the gland was to secrete this material from the blood, the lymphatics carrying it off. Baber[2] observed also the presence of red blood-corpuscles in different stages of disintegration and decolorization in the vesicles of one or both lobes in nine out of ten dogs examined. These corpuscles were sometimes few in number, and at other times completely filled the vesicle. He regarded the escape of red blood-corpuscles as almost constantly taking place into a greater or less number of vesicles, and that it was a normal occurrence.

In point of development the normal gland may show considerable variation even in the newly-born animal. Zielinska[4] found this to be the case when examining seven thyroids, six of which were from one litter of puppies. The difference depended essentially upon the degree of development of the colloid-containing vesicles. In some of the six there was an abundance of solid clusters of cells. In a few of these clusters a lumen could be distinguished with the aid of a powerful lens. In the others there was a more advanced type of development, half of the vesicles containing colloid, the remainder being either still in a condition of solidity or exhibiting only an empty lumen. A series of fourteen older animals, whose ages varied from four months to seven years, showed the vesicular development still further advanced, and in some instances complete disappearance of the solid clusters. The lymphatic channels also contained colloid.

ACCESSORY BODIES. In the immediate neighborhood of the glands, lying in the surrounding fascia, are a number of separate nodular bodies, among which may be noted by microscopical examination nodules of thymus tissue, masses of lymphoid tissue, and frequently nodules of true thyroid tissue, which have become detached from the main body at some period of their development, but have retained all its morphological character and probably also share in its function. The last-mentioned bodies are designated *accessory thyroids*. They are characterized by inconstancy of occurrence and uncertainty of position, being found at all levels, from the thyroid region to the arch of the aorta. L. R. Müller[5] and Kohn[6] have minutely described them. The latter authority also observed thymus nodules both within and without the thyroid lobes. Wölfler and Wagner[7] found accessory thyroids to be quite commonly present about the arch of the aorta in young dogs, occurring as single or double brownish-red bodies varying in size from a pinhead to a lentil. Their minute structure was identical with that of the thyroid. Halstead,[8] who carried out a great many thyroidectomy experiments, concluded from the evidence of his researches that these accessory bodies were almost invariably present. Ellenberger and Baum[1] have only been able to demonstrate their presence in the larger breeds, and these were situated in proximity to the gland. They were seldom able to find them about the aortal arch or in the hyoid region.

*The Thyroid Glandules or Parathyroid Glands.*

It is only within recent years that the identity of these bodies has been established. Henle,[9] describing the ductless glands in 1866, wrote the following significant sentences: "Of many of these we believe that we know the ultimate elements and their arrangement. But as long as the function, not only of these elements but also of the organs themselves, remains an unsolved problem one cannot but suspect that their anatomical structure contains some hidden secret."

W. Müller[10] seems to have been the first to notice that certain portions of the glands possessed a structure dissimilar to that of the remainder. Sandström[11] first described the glandules differentially in 1880, and about the same time Baber[2] referred to them as "undeveloped portions of the thyroid," stating that they consisted of solid masses of more or less cylindrical rows of cells. In dogs of three months and upward he usually observed them as distinct bodies not continuous with the normal gland tissue, but separated from it by layers of connective tissue, and frequently lying in a depression on the surface of the gland. He thought it remarkable that although these bodies were of frequent occurrence, there was usually in dogs aged three months and upward no evidence to show that they were undergoing further development.

In 1888 Rogowitch[12] recognized them, describing them as "embryonal remains" sharply defined from the rest of the thyroid tissue. In 1896 Vassale and Generali[13] published the results of their observations, showing that there are regularly present on each side two glandules instead of only one, as was believed by the earlier observers. Kohn[6] states that they are constantly present on each side as two bodies, one external to the gland and the other within the gland, there being sometimes a plurality of the external ones. The *external* show considerable variation of position, but usually one of proximity to the thyroids, and union of the two portions is quite exceptional. L. R. Müller[5] rarely failed to detect them with the naked eye. He described them as small superficial nodules about the size of a hemp-seed. Gley[14] examined their disposition in thirty-three dogs, and found that it was not always constant. In fourteen of the animals they were situated about the superior third of the external face of each lobe, nearer the anterior border than the posterior, superficially inserted in the face of but nevertheless perfectly distinct from the thyroid lobes. In seven of the remaining nineteen animals one glandule was enclosed in the external face, and the other isolated at the superior

FIG. 1.

FIG. 2.

FIG. 1.—Photograph of thyroid gland of full-grown Great Dane dog, showing (B) external glandule at superior extremity of gland. (Natural size.)

FIG. 2.—The same, showing opposite side of gland with outline of internal glandule (A) and external glandule (B). (Natural size.)

extremity of the corresponding lobe, being separated from this lobe by a tract of loose connective tissue. Thus this disposition was quite frequent. In one case one glandule was in the most common position, and the other at the middle of the external face of the other lobe. In another one glandule was in the most common position, the other at the inferior extremity of the external face. In another instance the glandules were in the ordinary position, but not enclosed, being attached by connective tissue to the capsule of each lobe. Twice both bodies lay isolated at the superior extremities of the lobes, and once both were isolated at the inferior extremities. These glandules receive each a twig of the ramifications of the thyroid arteries. Zielinska[4] found them almost constantly at the periphery of the superior extremities of the lobes, sometimes without, sometimes within the capsule, or in the vicinity of the entrance of the vessels. Moussu[15] wrote of frequently finding supplemental glandules on either side in the perithyroid or peritracheal connective tissue, or along the ramifications of the thyroid artery, and later speaks of extirpating such supplemental bodies. The *internal* are situated toward the internal or tracheal surface of the lobe, and as a rule are completely covered by thyroid tissue and regularly enter into extensive combination with it. Zielinska[4] states that they are much smaller than the external ones, and are found near the centre of the lobes. Structurally, neither of these

FIG. 3.

Diagrammatic sketches illustrating possible positions of the glandules.

bodies corresponds at any time of its development to the thyroid. They are epithelial bodies. Schmid[16] describes them as clusters of cells disposed, as a rule, concentrically around a small lumen or a globule of colloid matter. The importance of these glandules will be better appreciated after perusal of what is written in the remainder of this article.

### *Struma. Goitre. Bronchocele.*

Enlargements of the thyroid gland are quite common in the dog. We have already seen that they may be at times purely physiological. Such enlargements occur occasionally in puppies before birth, and may be of such dimensions as to hinder delivery (*S. congenita*). It is believed by some that heredity plays some part in its development, and Halstead's[17] experiments would seem to substantiate this view. In one case five puppies were born with thyroids developed to a size twenty times larger than the normal. The sire had been deprived of all of one lobe and two-fifths of the other, and the dam was in possession of only one. In another case, in which the dam had suffered removal of the left lobe and lower third of the right, and the sire possessed both intact, the puppies were born with glands twelve times as large as normal. The lobes and isthmus were so developed that they formed a single horseshoe-shaped body almost encircling the trachea. Pütz[18] saw two cases in Switzerland occurring in the same litter, and Wörz[18] one case in Germany in which the swelling disappeared later. Raynard[19] wrote in 1836 that it occurred among pointers and pugs in France. It has also been witnessed in puppies, the size of the thyroids of whose parents showed no deviation from the normal. Very young dogs are often seen with enlargements of the thyroids. Delabere-Blaine[20] state that it may commence soon after birth in members of certain breeds, mentioning Pomeranians, French pointers, dachshunds, spaniels, and lap-dogs. Greenhow[21] saw it in India, and Bramley,[22] writing in 1833, found it to be quite common as a "lobulated bronchocele," among puppies one month old bred from English dogs, in the trans-Himalayan regions. Van Gemmern and Mecke[23] say it seldom becomes large when arising in young dogs. According to Kitt,[24] it gradually becomes reduced in size, but in some instances will recur periodically.

In the experience of Zschokke,[25] it is a rare thing to find dogs free of thyroid enlargements in some of the cantons of Switzerland.

Several forms of struma are recognized. The enlargement may be the expression of extreme vascular engorgement (*Struma hyperœmica*). This is of a transitory nature and with little if any pathological significance. Mühlibach[26] observed that it occurred during the estrual period, and Bardeleben[18] saw it in pregnant bitches. Pflug[27] refers to a remarkable periodical recurrence in members of certain breeds, particularly Blenheim spaniels. The swelling appears coincidently with even a slight cold, but disappears within two weeks. Kölliker,[28] writing of vascular goitre, says that beside a hyperæmic condition there may be numerous aneurismal dilatations of the small bloodvessels. By the bursting of these apoplectic vesicles of different sizes are formed, and their contents become modified in color and consistence. Blood may also be extravasated into and infiltrate the connective tissue adjacent to the gland, or even of the entire length of the neck (*Struma hemorrhagica*). There is then seen diffuse swelling of the neck, with local pain and heat, which may or may not terminate in suppuration.

The commonest form is that of diffuse parenchymatous hyperplasia (*Struma hyperplastica, follicularis, simplex*), with or without a certain amount of cystic formation and proliferation of the stroma. Zielinska[4] studied this condition and found that the structure might be of alveolar type, composed of solid clusters of large cells, resembling hepatized lung, and showing no trace of colloid matter, but a necrotic central portion. In another case he observed that it might depend on augmentation and intercommunication of the colloid containing vesicles, with rich proliferation of the stroma. In the normal gland he had found colloid matter present in the lymphatic channels and occasionally in the veins as well as in the vesicles, but in struma both the arteries and veins might contain it, a part of their lumina still being free for the passage of blood cells. When these vesicles become considerably enlarged and confluent, fluctuating cysts are formed (*Struma cystica*). Kitt[24] says the contents of these cysts may undergo retrogressive metamorphosis with fatty degeneration, and blood may also be extravasated from ruptured vessels, so that on puncture the escaping matter may be yellowish, brownish, blood-stained, gelatinous, or resemble a milky emulsion. As Baber[2] found that the vesicles in the normal gland often contained red blood-cells and the products of the disintegration of the latter, an increase in the number of such cells might account for the discoloration of the cystic contents without actual rupture.

Sometimes thick septa of the interstitial connective tissue develop with consequent atrophy of the vesicles (*Struma fibrosa*).

A very rare form of osteochondroma (*Struma ossea*) has been observed by both Siedamgrotzky[29] and Kitt,[24] and I have also had the opportunity of witnessing the same condition in an aged collie bitch. There was a unilateral enlargement fully the size of the subject's own cranium. The glandular tissue had almost completely disappeared, a few minute isolated cysts and cell clusters marking the areas of functional persistence.

Malignant neoplasm (*Struma maligna*) not uncommonly affects old animals. It is of epitheliomatous or carcinomatous character, sarcoma never having been observed for a certainty, according to Kitt.[24] It tends to infiltrate neighboring structures and to lead to formation of secondary growths in the veins and in the lungs by way of the veins and lymphatics. It is distinguished from the other forms by its tuberculate character and by the cachexia which generally accompanies it.

The condition known as exophthalmic goitre is exceedingly rare. Coincident with the thyroid enlargement there are symptoms of puffiness of both eyes, retraction of the lids and extreme protrusion of the orbits, accompanied with corneal ulceration. Jewsejenko[30] saw this form accompanied with epileptiform paroxysms.

The effect on the organism of the different forms of goitre varies according to the nature, size, and position of the growth. Some of the largest simple goitres hardly affect the animal other than to render him unsightly. On the other hand, quite insignificant growths have been known to produce serious respiratory disturbances, with spasm of the glottis, owing to compression of the vagus and sympathetic nerves. Very voluminous goitres may induce suffocation by causing a narrowing of the lumen of the trachea and larynx. This is true also of the hemorrhagic form. Siedamgrotzky[29] saw the œsophagus completely encircled. Möller[30] has seen dogs with enormous goitres unable to lie down on account of the pressure on the trachea induced by that act. Van Gemmern and Mecke[23] saw vomiting (probably reflex) induced in a one-year-old Italian greyhound when the gland was enlarged, which, however, ceased when the swelling subsided. Cadeac[31] says laryngeal hemiplegia may result from pressure on the recurrent laryngeal nerve. Complete suppression of thyroidal function is followed by cretinism and myxœdema, conditions characterized by physical degeneracy and deformity and grave nerve disturbances. There occur an increase in the general connective tissue, with a mucoid conversion of the ground-substance, and marked idiocy. Rougieux[32] has recorded cases of cretinism, and Raynard[19] has seen the congenital form accompanied by imperfect development of the body and legs, thickened head, shortened neck, and feeble mental power. Experimental myxœdema and cretinism have been produced by Moussu[15] by complete extirpation of the gland, leaving the glandules intact.

Struma can be comparatively easily diagnosed. Generally the enlargement is bilateral, but not necessarily of uniform development. This bilateral character is of assistance in making a differential diagnosis from mucous cysts, abscesses, and hæmatomata. Furthermore, its mobility, sharp demarcation, and freedom from sensitiveness aid in the diagnosis. It can hardly be confounded with any other lesion unless it be lymphosarcoma involving the neighboring lymphatics, but in the latter disease other lymphatics are usually found to be involved. The enlargement may be so deeply embedded that its presence is hardly suspected, and in other cases may be so extensive as to occupy the entire distance between the trachea and sternum. Leisering[33] saw such a growth, it being an epithelioma, with secondary growths in the walls of the internal jugular.

The accessory bodies may also become enlarged, when they receive the name of "struma aberrantes." Wölfler and Wagner[7] found them to be as large as a pea in one instance in a two-months'-old dog possessing a congenital struma. It is not clear whether the enlargements in this instance were of nodules of true thyroid tissue or of the external glandules, as the report was made before the histological identity of the latter bodies had become established; but in another instance there was a veritable enlargement of a nodule of true thyroid tissue in an animal the lobes of which slightly exceeded the normal in size. The tumor was as large as a hazel-nut, and hung from the aorta by a pedicle.

The pathology of the glandules does not appear to have been studied up to the present.

Treatment. The simple forms (*S. hyperplastica* and *S. hemorrhagica*) generally respond to iodine medication (administered internally and by local inunction). I have seen exceedingly large simple goitres reduced by this method within a week. Failing in this, strictly aseptic intraglandular injections of the tincture of iodine may be tried. After the needle has been inserted one should first ascertain that its point has not lodged within the lumen of some enlarged vein, otherwise it must be partially withdrawn and then reinserted. The danger consists in the immediate entry of the iodine into the venous circulation. Horsley[3] experimentally injected 15 c.c. of tincture of iodine into the external jugular vein. Death was instantaneous from cardiac paralysis by plugging of the right heart with a hard clot.

Repeated injections at intervals of two or three days may be necessary.

Some idea of the manner in which the iodine becomes diffused throughout the gland may be gathered from the post-mortem investigations carried out by Baber.[2] He injected Berlin blue solution for staining purposes, and the following appearances presented themselves: The whole gland swelled up, and a fine network of injected vessels (lymphatics) appeared on the surface of the organ which could be distinctly seen with a hand lens. At the same time the lymphatic vessels running from the gland became injected.

Struma cystica is treated by free lancing of the sac and evacuation of the contents, but it must be remembered that the secreting membrane needs to be destroyed, which can be accomplished by iodine injections; otherwise a fistula is likely to be established. An antiseptic tampon is then introduced in order to stimulate healthy granulations. The malignant form being so extremely metastatic to important internal organs, and being usually accompanied by profound cachexia, scarcely warrants any attempt at giving relief even by surgical means. Unilateral neoplasm in the early stages might perhaps justify unilateral extirpation or complete extirpation, provided the glandules were healthy and left *in situ.*

A case of exophthalmic goitre[34] was treated by local disinfection and inunction of belladonna and iodine, and supplemented by injections of tincture of iodine into the gland, with cold baths. It terminated in complete recovery. It is worthy of note that this form of struma can be cured in the human subject by partial removal of the thyroid (Wharton and Curtis[35]).

### *Thyroidectomy.*

Until recent years and the discovery of the thyroid glandules, it was generally believed that ablation of both lobes of the gland was necessarily followed by fatal results within from three to twenty-one days. Of sixty-three total extirpations of healthy gland by Halstead,[8] all the subjects died. Schiff [36] excised the gland *in toto* from sixty dogs, and found the animals died within from three to twenty-seven days thereafter, with but one exception. Death was preceded by symptoms of indifference, melancholia, great itching of the skin, clonic and tonic muscular spasms, fall of blood-pressure from vasomotor paralysis, and purulent conjunctivitis, with corneal ulceration. To these symptoms Gley[37] adds those of vomiting and salivation, which occurred daily until the animal's death; anorexia, accelerated respiration, hyperpyrexia, and albuminuria. These results were more rapid in young animals. In Schiff's exceptional case the animal had survived nearly fifty days at the time of the report, and seemed likely to make a permanent recovery. Beside Schiff, several other experimenters have observed that thyroidectomy is not invariably followed by death. Zesas[38] recorded a case in which the animal seemed to have recovered. Albertoni and Tizzoni[39] had four recoveries in twenty-four cases; Fuhr[40] one in fourteen; Bogowitch[41] four in forty, and Artland and Magon[42] one in four. The latter observers found nodular bronchopneumonia, hepatitis, and nephritis as lesions in the three animals that succumbed.

Halstead[8] carried out eighty-eight transplantation experiments but without a single successful outcome, as the transplanted lobes were always quickly absorbed. Schiff[36] placed a freshly excised thyroid of one dog in the abdomen of another, waited from two to five weeks to let it become engrafted (?), and then removed both lobes of the latter's thyroid at one operation, and found that the animal might survive.

Halstead[8] found that unilateral extirpation of healthy glands was followed with good results, the remaining lobe undergoing hypertrophy within forty days of the time of removal of the other. Schiff[36] observed that if the lobes, instead of being both removed at one operation, were removed at two operations, separated by a considerable interval (for adults twenty days), it was possible to keep the animal alive. This he accounted for by supposing the function of the gland passed to accessory organs, but only very slowly. It is most probable that all the instances of survival after complete extirpation of both lobes were due, not to any substitutional functioning on the part of the accessory thyroids, as most of the experimenters believed, but rather to undisturbed continuance *in situ* of isolated or detached external glandules. Moussu[15] has advocated this explanation. During some of his investigations he found supplemental glandules. In one animal there were two glandules on the right side, one being situated on the external surface of the lobe, the other isolated at the superior extremity. On the left side one was isolated at the point of entry of the superior thyroid artery, while two others were situated in the peritracheal connective tissue, each supplied with a ramification of the thyroid artery. Before the presence of the internal glandules had become known, Gley[37] removed the thyroid and left the two external glandules intact. He performed this somewhat delicate operation in ten cases, of which two died. One was sick at the time of the operation, and evidently died from tuberculosis, and in the other only one glandule had been left intact. The remainder of the animals showed only slight transient symptoms. He considered that removal of the thyroid alone was innocuous. In these operations he had, of course, unknown to him, removed the internal glandules. When the external glandules alone were removed the operation was productive of no result. Cadéac and Guinard,[43] Paladino,[44] and Capobianco[45] made similar observations.

Moussu[15] performed eight thyroidectomies. Two of the animals died. In one of them only one glandule had been left intact, and the other probably succumbed, owing to injury sustained by the delicate parathyroid veins, thus interfering with the function of these bodies. Unlike Gley, this observer found the animals became the subjects of myxœdema and cretinism when two glandules were left intact. Within a week they became depressed in spirit, the face wrinkled, and they acquired a general aspect of age. The feet shortened, the trunk enlarged, the abdomen became rounded, and the skin infiltrated and thickened. He advises that at least two of the glandules, and preferably three or four, should be left intact when extirpating the thyroids. Vassale and Generali[46] extirpated all the glandules in nine dogs, and all died within eight days. The symptoms were very similar to those which follow complete thyroidectomy, which had hitherto meant removal of both glands and glandules. They differed in that the convulsive phenomena were only very slightly manifested, and, as a rule, just previous to death, whereas paralytic symptoms were prominent. In another series of experiments they varied the operation in a number of ways. Removal of both glandules on one side produced no morbid phenomena in three cases, and only slight transitory symptoms in the fourth. Extirpation of the two remaining glandules of the other side some time after removal of the first pair resulted fatally in from five to thirteen days. Extirpation of the two internal glandules was followed by no morbid phenomena. Subsequent extirpation of the two external ones resulted in death in from three to four weeks. Removal of three glandules, an internal one being left, produced only transitory symptoms. Subsequent removal of this glandule, together with the lobe of the thyroid with which it was associated, resulted in death in twelve days. Removal of the thyroid lobe on one side, and one external glandule on the other side, produced no morbid symptoms. Extirpation of the entire thyroid, leaving *in situ* only two external glandules, caused slight symptoms in one dog and none in three others. Complete removal of the thyroid, one external glandule being left, caused transient symptoms in one case and none in another. They concluded that suppression of the function of the thyroid gland produced only myxœdema, which agrees with Moussu's observations. In fatal cases where the glandules had been removed there were degenerative changes in the nervous system, particularly of the spinal cord. Edmunds[47] concludes that if the whole of one lobe of the thyroid, including its external glandule and also the greater part—two-thirds or more—of the other lobe, be removed the animal will live or die according as the external glandule is or is not left.

Halstead found (apparently before knowing of the importance of the glandules) that piecemeal excision of both lobes (healthy) by successive operations, when the reduction was not carried much beyond three-fourths of each lobe, did not prevent the animals from enjoying good health.

It becomes then necessary, in discussing operative measures on the thyroid gland, to speak of unilateral and complete thyroidectomy, and unilateral and complete external and internal parathyroidectomy or extirpation of the thyroid glandules.

To what extent these respective operations may be undertaken as curative measures for goitre cannot yet be stated with any degree of certainty. That is a matter which must depend altogether on the results of clinical investigations and a knowledge of the pathological conditions to which the glandules may be subject. So far as I have been able to ascertain, it is not yet known whether changes in the glandules occur concurrently with those taking place in a goitrous gland or not.

It is probable that unilateral extirpation of both gland and glandules can always be undertaken with safety if we have good reason to believe that the corresponding organs on the opposite side are still capable of functioning.

Unilateral Thyroidectomy, without regard to conservation of the glandules, is carried out as follows: Make the skin incision in the median line. This enables the operator to get down easily between and without severing the muscles, which is conveniently done by tearing with the finger or with the aid of a blunt instrument. The lobes are found one on each side under the sternothyroid muscles. Their mobility and slipperiness make their removal somewhat difficult. Draw the lobe up out of the wound by means of a suture passed through it, and secure the ramifications of the superior thyroid artery with a ligature, including the tissue surrounding them, apply another ligature around the anastomosing termination of the inferior thyroid, and, lastly, divide all the attachments on the distal side of the ligatures, leaving as small a stump as possible. It is worthy of note that the necessity of maintaining an aseptic wound in thyroid operations was particularly emphasized by Munk[48] in his experiments, and latterly by Halstead, who found it expedient to devise his "subcuticular suture."

Simple Thyroidectomy, leaving the glandules intact, is thus described by Gley[15] (translation): When the glandules are isolated at the superior or inferior extremity of the gland the operation is not difficult. But this disposition is not the most frequent, consequently it is often necessary to explore for and enucleate them from the thyroid body. Secure the superior and inferior extremities of the lobes by two separate sutures. One of these sutures may often be made to include the thyroid artery, but it is particularly essential that the minute vessel which detaches itself to furnish the glandule be left free. By means of these two sutures have an assistant draw up the lobe in such a manner as to render the glandule visible. Separate the latter little by little from the adjacent tissues with a blunt instrument. Now pass a fine ligature behind it, but in such a manner as not to include a veinlet which receives branches from the lobe at this level. If necessary a portion of the lobar tissue may be included. Finally, remove the lobe. There is only a slight oozing of blood during the operation.

Moussu found it very difficult to preserve the veinlets, which are necessary for the proper performance of the parathyroid function.

Gley and Nicolas[49] found that the glandules underwent hypertrophy after extirpation of the gland.

Breisacher[50] noticed that dogs fed on raw meat suffered more acutely from thyroidectomy than those fed on milk and boiled meat.

### Bibliography.

1. Anatomie des Hundes.
2. Philosophical Transactions of the Royal Society of London, 1876, p. 557 ; 1881, p. 583.
3. British Medical Journal, 1885, p. 213.
4. Virchow's Archiv, 1894, p. 136.
5. Beiträge z. Pathol. Anat. u. z. Allg. Pathol., Jena, 1896.
6. Archiv f. Mikr. Anat., 1895, 1896, 1897.
7. Wiener med. Wochenschrift, 1879, p. 198.
8. Johns Hopkins Hospital Reports, vol. i.
9. Baber's Translation from Handbuch d. System. Anat. des Menschen, vol. ii., 1866, p. 35.
10. Jenaische Zeitschrift f. Medizin u. Naturwissenschaft, 1871.
11. Lakareförenings Fördhandlingar, Upsala, 1880.
12. Archiv de Phys. Norm. et Path., 1888.
13. Rivista di Patol. Nerv. e Ment., 1896.
14. Comptes-rendus de la Soc. de Biologie, 1893, pp. 217 and 396.
15. Comptes-rendus de la Soc. de Biologie, 1893, p. 394 : 1897, p. 82.
16. Archiv f. Mikr. Anat., 1896.
17. Johns Hopkins Hospital Bulletin, vol. i.
18. Cited by Pflug in Deutsche Zeitschrift f. Thiermed., 1875, p. 340.
19. Rec. de Méd. Vétér., 1836, p. 8.
20. Handbuch. ü. d. Krankheiten der Hunde, p. 214.
21. Indian Annals of Medical Science, 12.
22. Transactions of the Medical and Physiological Society of Calcutta, 1833, p. 195.
23. Anweisung z. Vorbeng. u. Heilung d. Krankheiten d. Hunde, p. 55.
24. Lehrbuch. d. Patholog.-Anatom. Diagnostik, 2.
25. Schweiz. Archiv. f. Thiermed., 188. p. 52.
26. Der Kropf, 1822.
27. Deutsche Zeitschr. f. Thiermed., 1875, p. 349.
28. Handbuch d. Gewebelehre, 5th ed., 1867.
29. Bericht. u. d. Veterinärwesen im Königr., Sachs, 1871, p. 58.

30. Cited by Kitt in Lehrb. d. Patholog.-Anat. Diagnostik, 2.
31. Patholog. Interne des Animaux Domestiques.
32. Cited by Morel in Ann. Méd. Psych., 1874. Köberle in Essai sur le Crétinism. Strassburg, 1862, p. 38.
33. Bericht u. d. Veterinärwesen im Königr. Sachs, 1872, p. 59.
34. Journal of Comparative Medicine and Surgery, October, 1888.
35. Practice of Surgery.
36. Rev. Méd. de la Suisse Romande, February, 1884.
37. Archiv. de Phys. Norm. et Pathol., 1892, p. 81; 1893, p. 767.
38. Archiv. f. klin. Chirurg., 1884.
39. Archiv. per le Scienze Mediche, 1886, p. 45.
40. Archiv. f. Exper. Path. u. Pharmak., 1886.
41. Archiv. de Phys., 1888, p. 419.
43. Comptes-rendus de la Soc. de Biolog., 1894, p. 468.
44. Atti Della Reale Accad. Med. Chir. di Napello, 1893.
45. La Riforma Medica, 1895.
46. Rivista di Patolog. Nervosa e Mentale, vol. i. p. 95. Archiv. Italien. de Biolog., 1896.
47. Proceedings of the Royal Society, 1895–1896.
48. Cited by Halstead in Johns Hopkins Hospital Reports, vol. i.
49. Comptes-rendus de la Soc. de Biolog., 1895. p. 218.
50. Archiv. f. Anat. u. Phys., 1890.

## Figures and Tables

**Fig. 1. Fig. 2. f1:**
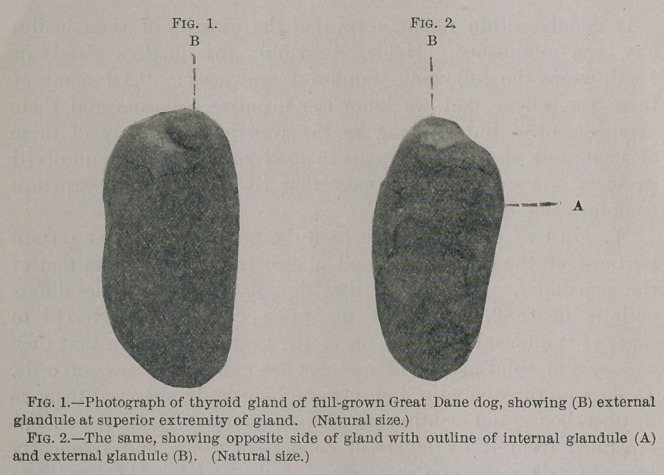


**Fig. 3. f2:**